# Diversity in the editorial boards of global health journals

**DOI:** 10.1136/bmjgh-2019-001909

**Published:** 2019-10-18

**Authors:** Soumyadeep Bhaumik, Jagnoor Jagnoor

**Affiliations:** 1 The George Institute for Global Health, India, New Delhi, India; 2 The George Institute for Global Health, University of New South Wales, Sydney, New South Wales, Australia

**Keywords:** public health

**Summary box**

- We present a new scoring system (Composite Editorial Board Diversity Score (CEBDS)) to evaluate the diversity of editorial board in terms of three parameters—gender, country income-level and geographic region.
- We analysed the diversity of the editorial boards of 27 specialty global health journals—of 303 editors, 40% were females; 68% based in high-income countries; 34% were based in Europe and Central Asia and 30% were based in North America. And among editors-in-chief, 27% were females and 73% were based in high-income countries.
- Only 26% of journals had the highest possible score in the gender diversity domain (40%–60% female editors), 11% had the highest possible score in the country income-level domain (at least one editor in all country income groups) and 7% had the highest possible score in the geographic region diversity domain (at least one editor in all six regions). Overall, only 11% of journals had high CEBDS (≥8).
- There is need for studies to understand enablers and barriers of diversity in journal editorial boards. Affirmative action and application of organisational good practices for improving diversity, inclusion and belongingness is required to ensure diversity in editorial board of global health journals.

## Introduction

Diversity in global health workforce and leadership has received a lot of attention, particularly in terms of gender and representation of low/middle-income countries in global health institutions like the WHO.^1–4^ Representation of women in medical journals has been studied extensively including for authorship, peer-reviewers and editorial positions in several medical specialities,^5–14^ with only a few studies analysing geographical diversity.^15 16^ Although many journals champion diversity narratives in several domains of global health,^17–19^ the issue of diversity in specialty global health journals has not been studied previously.

Having an editorial role in an academic journal reflects status and indicates leadership and influence within a field. Thus, having diverse editorial boards can help to promote diverse and balanced perspectives, ensure equity and fairness, serve a role modelling function for future generations and enable the decolonisation of global health research evidence and narratives. In addition, a diverse editorial board can also provide access to a wider pool of peer-reviewers and encourage submission from researchers of diverse backgrounds. We therefore sought to understand the diversity in editorial board of specialty global health journals.

We identified 27 specialty global health journals (from the National Library of Medicine, USA catalogue^20^—see online supplementary file for search terms, inclusion criteria and process). We analysed their editorial boards for diversity in 2018. To assign diversity scores, we developed a Composite Editorial Board Diversity Score (CEBDS) which evaluated diversity in three domains: gender, country income-level and geographic region. Scores were assigned in each domain as in table 1 and CEBDS was calculated by adding up all the individual domain scores. Journals were considered as having poor diversity if CEBDS was ≤5, moderate diversity if CEBDS was 6 or 7 and good diversity if CEBDS was ≥8.

10.1136/bmjgh-2019-001909.supp1 Supplementary data

**Table 1:** Scoring systems for different domains leading to the calculation of CEBDS

Score assigned	Gender diversity domain (GID)	Country income-level diversity domain (CIDD)	Geographic region diversity domain (RID)
0	All male or all female editors	All editors based in one World Bank Income classification group	All editors based in one World Bank Region
1	Not applicable	At least one editor based in two of the four World Bank Income classification groups	At least one editor based in two or three of the total seven World Bank Regions
2	1%–39% female editors	At least one editor based in three of the four World Bank Income classification groups	At least one editor based in four or five of the total seven World Bank Regions
3	Not applicable	At least one editor based in all four World Bank Income classification groups	At least one editor based in six of the total seven World Bank Regions
4	40%–60% female editors	Not applicable	Not applicable
CEBDS calculation	GID + CIDD + RID (minimum score 0; maximum score 10)		

CEBDS, Composite Editorial Board Diversity Score.

We determined gender (binary), using a sequential approach (ie, by going to the next criterion if the preceding one did not yield enough information): first, editor description on journal website; then a web application, Genderize,^21^ which determines the gender of a first name along with a probabilistic certainty score (accepted only when ≥0.95); then editor description on institutional website; and finally, inspection of names by authors (decided by consensus). We also used a sequential approach to identify the country in which editors are based: first, editor descriptions on the journal website; then editor descriptions on institutional website. For gender and country, if none of the steps led to an inference, we marked the category as unclear for the editor.

We grouped countries using the World Bank Classification^22^ to categorise by region (seven groups: East Asia and Pacific, Europe and Central Asia, Latin America and the Caribbean, Middle East and North Africa, North America, South Asia and Sub-Saharan Africa) and income status (four groups: low-income, lower-middle-income, upper-middle-income and high-income economies).

## Diversity in editorial boards of global health journals

All but one of the 27 journals are published in high-income countries—that is, *Clinical Epidemiology and Global Health,* published in India. Of the 303 editors listed on the websites of the 27 journals, there were 122 female (40%) and 168 male (56%) editors; and the gender of 13 (4%) editors could not be determined based on the available information. However, only 10 out of 37 (27%) editors-in-chief were females. Most managing editors were females (78%—ie, 14 out of 18). Among female editors most were associate editors (57%—ie, 70 out of 122), but women were only 39% of associate editors (70 out of 181)

The country of location was not clear for 23 editors (8%). But the majority of editors are based in high-income countries (68%, n=206). Among editors-in-chief, 73% (27 out of 37), are based in high-income countries and none in low-income countries and only one journal had an editor based in a lower middle-income country. Regionally, 30% (n=91) of editors are based in the North America, 34% (n=102) based in Europe and Central Asia and only 2% (n=6) based in the Latin America and Caribbean region. The distribution of editors-in-chief across different regions was similar, but there were no editors-in-chief based in the Latin America and Caribbean region (see online supplementary file for detailed information on individual domains and editorial position subtypes).

**Figure 1:**
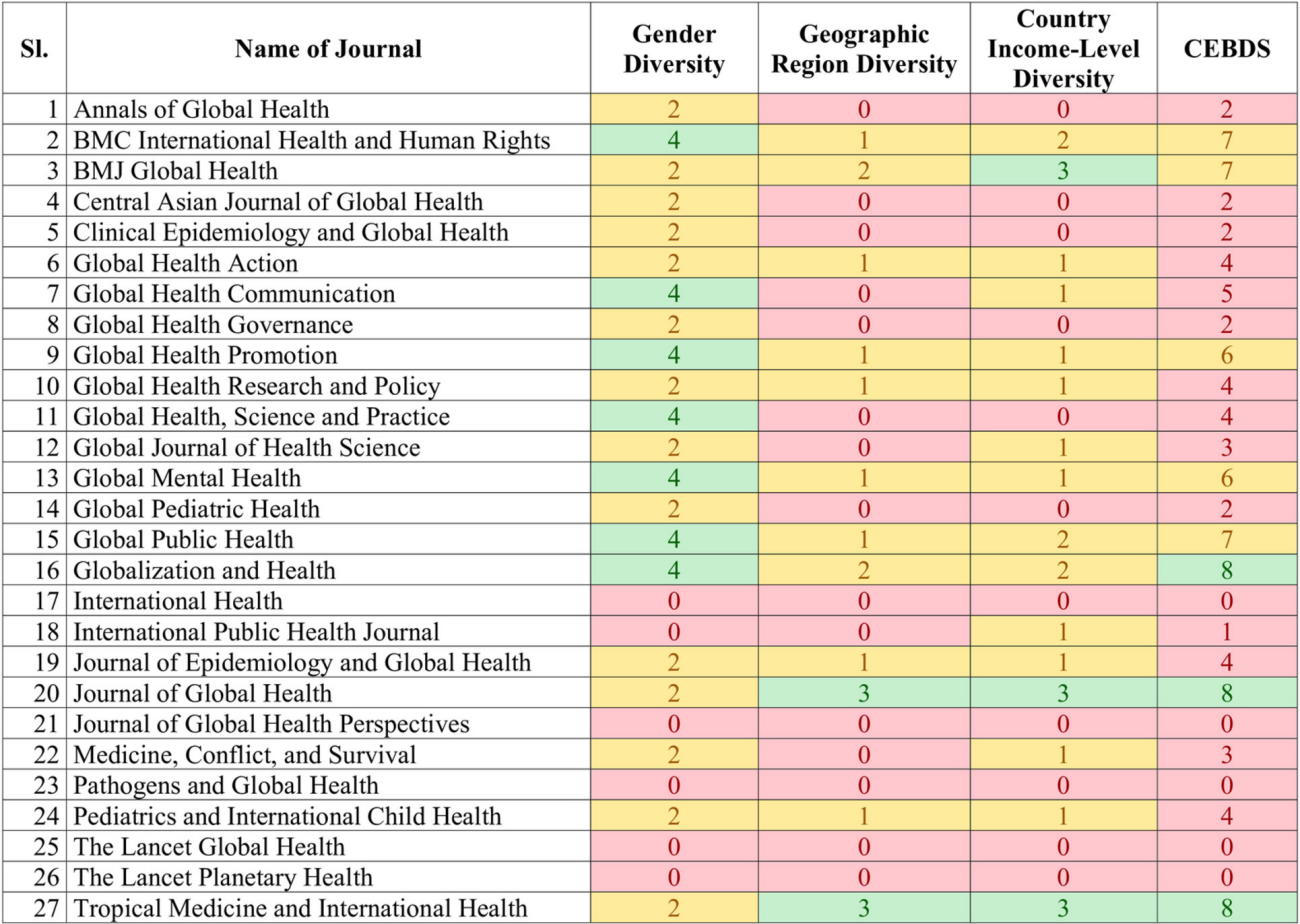
Diversity scores of specialty global health journals. CEBDS, Composite Editorial Board Diversity Score.

The scorings for individual domains, as well as the overall CEBDSs are presented in figure 1. The highest possible diversity score for individual CEBDS domains was achieved in only three journals; and none got the highest possible score in all the three domains. Only seven journals had the highest score in the gender diversity domain, three had the highest score in the country income-level domain and two had the highest score in the geographic region diversity domain. Five journals got the lowest possible score of 0 indicating no diversity in their editorial boards.

We did not consider the existence of other gender identities (beyond the male–female binary) and acknowledge small possibilities of individual gender misclassification. However, we contend that these limitations are not likely to influence our findings. We developed the CEBDS as an objective measure of diversity for use on publicly available data—to allow comparison between journals and monitor progress over time without survey data from individual journal editors. It is important to understand diversity from a broader perspective including characteristics such as skin colour, class, socioeconomic status, sexual preferences, profession as well as social networks, especially of editors from low and middle income country (LMIC). However, such data are difficult to compile and may not be suitable for rapid scans or comparison across journals which CEBDS allows.

## Conclusion

Many global health actors are working to improve women representation in leadership positions, including at the United Nations and its agencies.^5 17 23 24^ But, as our findings indicate, the need for diversity in global health is not limited to gender. We noticed a ‘glass ceiling’^25^ effect for all three parameters—gender, geography and country income-level. The glass ceiling effect was such that, barring few exceptions, editors-in-chief are males based in high-income European or North American countries.

Existing studies show that key factors limiting the role of women in leadership positions are organisational gendering process and social barriers due to gender roles.^23^ But not much is known about barriers to having journal editors based in underrepresented regions of the world, and from low/middle-income countries. Academics in these settings may face unique challenges. Clearly, global health journals can do much better on diversity in their editorial boards, and should adopt affirmative action policies and organisational good practices on diversity, inclusiveness and belongingness.^26^
