# Supplementary material for: Diversity in the editorial boards of global health journals
Source: BMJ Glob Health. 2019 Oct 18;4(5):e001909. doi: 10.1136/bmjgh-2019-001909 (PMC6830044; doi:10.1136/bmjgh-2019-001909)
Supplement: Supplementary data [file bmjgh-2019-001909supp001.pdf]

Supplementary Appendix: Diversity in the Editorial Boards of Global Health Journals

Soumyadeep Bhaumik [1*], Jagnoor Jagnoor[1]

[1]The George Institute of Global Health, New Delhi, India

Contents

Eligibility criteria for inclusion of specialty global health journals 1

Identification of specialty global health journals 1

Detailed information on diversity of editors of specialty global health journals 2

Ethical considerations 2

Eligibility criteria for inclusion of specialty global health journals

Any scientific journal which is a specialty global health journal (global health being defined as per the definition of National Library of Medicine (NLM), USA Catalogue) was eligible for inclusion. We excluded any journal that has discontinued publication as per NLM record or did not list name of editors in their website.

Identification of specialty global health journals

In September 2017 , We identified global health journals through the National Library of Medicine (NLM), USA Catalogue with the following search code:

"Global Health"[Mesh] OR ("Public Health"[Mesh] AND "Internationality"[Mesh]) OR "global health"[All Fields] OR "international health"[All Fields] OR "international public health"[All Fields] AND ncbijournals[All Fields]" .

Two authors independently screened the records for inclusion as per eligibility criteria. Disagreements on eligibility were resolved by consensus discussion between two authors.

We retrieved 71 journals from our search in the NLM catalogue. Based on our criteria of specialised global health journal, 27 journals were included for analysis. Detailed rationale

Bhaumik S, Jagnoor J. *BMJ Global Health* 2019; 4:e001909. doi: 10.1136/bmjgh-2019-001909

for inclusion, list of all excluded journals and list of included journals is available from **Extended Data Set 1 in https://doi.org/10.6084/m9.figshare.9205340.v1**

Methods for data collection for composition of editorial board

We obtained information pertaining to all editors and their roles from their respective journal websites between October-December 2017. We did not collect information about those listed as editorial team/board members marked as consultants/ advisors/ methodologists/ support team or any other role that were not designated specifically as an editor. Data from https://genderize.io/ was collected from January to April 2018 .

Detailed information on diversity of editors of specialty global health journals

Detailed information on gender, income classifications and regions segregated by different editorial roles is reported in **Extended Data Set 2: https://doi.org/10.6084/m9.figshare.9205340.v1**

Ethical considerations

Since only data available in public domain (from journal and institutional websites) was used and study did not involve any living participant ethical clearance was not required. We did not use any data from social media, where the distinction between public and private domains is not so clear.

Bhaumik S, Jagnoor J. *BMJ Global Health* 2019; 4:e001909. doi: 10.1136/bmjgh-2019-001909
